# Prediction of Liver Triglyceride Content in Early Lactation Multiparous Holstein Cows Using Blood Metabolite, Mineral, and Protein Biomarker Concentrations

**DOI:** 10.3390/ani12192556

**Published:** 2022-09-24

**Authors:** Ryan S. Pralle, Henry T. Holdorf, Rafael Caputo Oliveira, Claira R. Seely, Sophia J. Kendall, Heather M. White

**Affiliations:** 1 School of Agriculture, University of Wisconsin-Platteville, Platteville, WI 53818, USA; 2 Department of Animal and Dairy Sciences, University of Wisconsin-Madison, Madison, WI 53706, USA

**Keywords:** fatty liver, transition cow, partial least squares, blood biomarkers

## Abstract

**Simple Summary:**

Bovine fatty liver syndrome is a metabolic disorder in transition dairy cows that has been associated with adverse consequences such as lower milk production and fertility. Fatty liver syndrome is difficult to monitor and diagnose in applied practice and research settings because it requires a liver tissue biopsy to determine liver triglyceride content. This study aimed to develop and validate a panel of blood metabolite, protein, and mineral biomarkers as a less invasive and more accessible tool to assess liver triglyceride content. We investigated a variety of panels using blood measurements from a single timepoint or multiple timepoints, as well as different combinations of biomarkers based on their perceived accessibility. Both the single and multiple timepoint biomarker panels accurately classified cows with high liver triglyceride content (top 33.3% vs. lower 66.7%), but accuracy was lower for classifying cows with or without maximum liver triglyceride in the top 50% or top 66.7% of liver triglyceride content. We suggest that the blood biomarker models predicting high triglyceride content may be useful for monitoring fatty liver in research and applied practice, as well as enable larger scale research studies investigating fatty liver in dairy cows.

**Abstract:**

Bovine fatty liver syndrome (bFLS) is difficult to diagnose because a liver tissue biopsy is required to assess liver triglyceride (TG) content. We hypothesized that a blood biomarker panel could be a convenient alternative method of liver TG content assessment and bFLS diagnosis. Our objectives were to predict liver TG using blood biomarker concentrations across days in milk (DIM; longitudinal, LT) or at a single timepoint (ST; 3, 7, or 14 DIM), as well as different biomarker combination based on their perceived accessibility. Data from two separate experiments (*n* = 65 cows) was used for model training and validation. Response variables were based on the maximum liver TG observed in 1 and 14 DIM liver biopsies: Max TG (continuous), Low TG (TG > 13.3% dry matter; DM), Median TG (TG > 17.1% DM), and High TG (TG > 22.0% DM). Model performance varied but High TG was well predicted by sparse partial least squares—discriminate analysis models using LT and ST data, achieving balanced error rates ≤ 15.4% for several model variations during cross-validation. In conclusion, blood biomarker panels using 7 DIM, 14 DIM, or LT data may be a useful diagnostic tool for bFLS in research and field settings.

## 1. Introduction

Bovine fatty liver syndrome (bFLS) is a metabolic disorder in early lactation dairy cows that develops in part due to negative energy and nutrient balance, obesity, and inflammatory signals [1,2,3,4]. Subclinical and clinical cases of bFLS are characterized by an abnormally high concentration of liver triglyceride (TG) in early postpartum cows, even though a diagnostic threshold has not been formalized [2]. A cross-sectional epidemiology study of 218 Dutch cows found 54% of the cows experienced a moderate to severe accumulation of liver TG between 6 and 17 days in milk (DIM) [5]. Several unfavorable performance outcomes have been associated with increased liver TG content in early postpartum cows, including lower feed intake, lower fertility, greater weight loss, and greater incidence of disease comorbidities [6,7,8]. The absence of pathognomonic clinical signs of bFLS has made TG quantification from liver tissue biopsy samples the standard for bFLS diagnosis. Liver biopsies are an invasive surgical procedure, which has likely limited their use in research and field settings. Limited implementation of biopsy-based liver TG quantification has in turn limited evidence for diagnostic thresholds, case prevalence, and consequences for bFLS. In contrast, much more is known about prevalence, outcomes, and diagnostic thresholds for other early lactation metabolic disorders such as hyperketonemia, which has multiple convenient, less-invasive tools for cowside diagnosis [2,8,9]. This dichotomy underscores the importance of developing a less invasive bFLS diagnostic approach.

Blood collection for assessment of early lactation dairy cows is not uncommon for other metabolic disorders like hyperketonemia and hypocalcemia [9,10,11] and may represent an opportunity to assess liver TG content or functionality. However, the previously mentioned *-emia* disorders are defined by quantification of a blood biomarker, whereas bFLS is defined by a liver tissue measurement. Thus, a diagnostic approach exploring the use of multiple blood biomarkers related to liver metabolism and functionality is likely prudent. Maladaptation to negative energy and nutrient balance is part of the gross pathology of bFLS; biomarkers of energy and nutrient status like non-esterified fatty acids (FA), β-hydroxybutyrate (BHB), and glucose are of particular interest and were previously associated with liver TG content [8,12,13,14]. Other indicators of liver protein and lipid metabolism associated with bFLS are blood urea nitrogen (BUN) and cholesterol, respectively [2,5,13]. Additionally, biomarkers of liver damage and the acute phase response, such as alanine transaminase (ALT) and aspartate transaminase (AST), albumin, and haptoglobin (Hp) are associated with liver TG in dairy cows [2,15,16]. While less explored in dairy cattle, blood mineral concentrations are associated with steatosis in humans and rodents, including calcium (Ca), phosphorous (Phos), and magnesium (Mg) [17,18,19]. Total blood calcium was previously associated with liver TG content in dairy cows [8]. An index of various blood biomarker concentrations has been proposed to indirectly assess cow liver activity and health [20,21] but prior attempts were validated based on cow milk production, reproduction metrics, and morbidity [20,21] rather than directly based on liver TG content or other relevant metrics.

We hypothesized that liver TG content can be directly predicted by a blood panel of metabolite, mineral, and protein biomarkers, providing a more convenient tool to diagnose and monitor bFLS and cow metabolic health. Leveraging blood energy metabolite, protein, and mineral concentrations analyzed on published data sets [22,23], the objectives of this research were to predict liver TG content in multiparous Holstein dairy cows using longitudinal (LT) or single timepoint (ST) blood sampling, and to improve the accessibility of these liver TG prediction panels by exploring combinations of explanatory variables based on the anticipated availability of samples or lab chemistry to most research and diagnostic labs. The selection of blood biomarkers was based on a combination of biological justification and practicality and resulted in measurement of albumin, ALT, AST, BHB, BUN, Ca, cholesterol, FA, glucose, Hp, Mg, and Phos. It is important to note that although other potential markers could be justified biologically, we refrained from using biomarkers that would not be practical for the final application of applied prediction models; therefore, we did not include biomarkers that can only be quantified by an enzyme-linked immunosorbent assay or radioimmunoassay.

## 2. Materials and Methods

Experiments conducted on the cows in this research followed animal use and handling protocols approved by the University of Wisconsin–Madison College of Agriculture and Life Sciences Animal Care and Use Committee (protocol A005467). All cows were housed in a tie-stall facility, at the Dairy Cattle Instruction and Research Center (University of Wisconsin–Madison, Madison, WI, USA).

### 2.1. Experimental Design, Sampling, and Analysis

Samples and data from multiparous Holstein cows (*n* = 65) used in this research are sourced from two published experiments [22,23]. A brief description of experimental treatments and pertinent sampling are provided below. Descriptive statistics of cow production and performance data are reported in Supplementary Table S1. The exclusive use of multiparous cows for this research was intentional due to their greater risk for metabolic disorders [24,25].

Experiment 1 enrolled multiparous Holstein cows (*n* = 40) and randomly assigned cows to treatment within block (expected parturition date). The treatments included a control diet (*n* = 20 cows) and a treatment diet supplemented with fermented ammoniated condensed whey (*n* = 20 cows, 2.9% diet dry matter (DM), replacing soybean meal). To increase risk of hyperketonemia cases, cows within both treatments were dietarily challenged by a daily top-dress of 6 kg of dry, cracked corn, in addition to ad libitum access to their total mixed ration (1.69 Mcal NE_L_ per kg DM), from -28 expected days relative to calving until parturition [22].

Like experiment 1, experiment 2 enrolled multiparous Holstein cows (*n* = 25) by random assignment of cows to treatment within block (expected parturition date). Treatments included a control diet (*n* = 13 cows) and a ketosis induction protocol (*n* = 12 cows). Prepartum, both treatments were allowed ad libitum access to a total mixed ration (1.42 Mcal NE_L_ per kg DM) and both treatments had the same postpartum diet. Cows within the ketosis induction protocol treatment were dietarily challenged by a daily top-dress of 6 kg of dry, cracked corn, in addition to the total mixed ration, from -28 expected days relative to calving until parturition. Additionally, the ketosis induction cows were feed restricted to 80% of voluntary intake beginning at 14 DIM [23]. All samples and data used in the present research were collected prior to the feed restriction period. 

In both experiments, blood samples were collected by venipuncture of the coccygeal vessels into evacuated tubes with or without additive at 1, 3, 5, 7, and 14 DIM before the daily feeding. Serum was separated from blood collected in tubes without additive (BD Vacutainer, Franklin Lakes, NJ, USA) after centrifugation at 2500× *g* for 15 min at room temperature. Plasma was separated from blood collected in an evacuated tube containing potassium oxalate and 4% sodium fluoride (BD Vacutainer, Franklin Lakes, NJ, USA) that was kept on ice until centrifugation at 2000× *g* for 15 min at 4 °C. Serum and plasma aliquots were stored at − 20 °C until metabolite, protein, and mineral biomarker analysis.

Details on some of the metabolite and protein biomarkers quantified have been detailed previously [22,23,26]. Blood fraction (serum or plasma) concentration of FA, glucose, and BHB were determined on samples collected on 1, 3, 5, 7, and 14 DIM. In experiment 1, plasma FA (Wako NEFA-HR(2) Microtiter Procedure kit; Wako Diagnostics, Richmond, VA, USA), BHB (Stanbio BHB LiquiColor kit; Stanbio Laboratory, Boerne, TX, USA), and glucose (Autokit Glucose; Wako Diagnostics) were quantified by plate assay [22]. In experiment 2, plasma FA was quantified by plate assay [23], while plasma glucose and serum BHB were quantified using the previously validated methods on a Catachem Well-T AutoAnalyzer (Catachem, Awareness Technologies, Oxford, CT, USA) [23]. Blood fraction (serum or plasma) concentration of BUN, albumin, ALT, AST, and Hp was quantified for samples collected on 1, 3, and 14 DIM. Across both experiments, BUN, albumin, ALT, and AST were quantified in plasma using the Catachem Well-T AutoAnalyzer (Catachem, Awareness Technologies) [26].

The following biomarkers were not previously reported. Serum Hp was quantified in triplicate based on a published assay [27]. Briefly, the difference in the peroxidase activity of the haptoglobin-hemoglobin binding complex was measured colorimetrically at 450 nm using a Synergy H1 Hybrid Spectrophotometer (BioTek, Winooski, VT, USA) using a 5-point serial diluted (1:2) standard of a known Hp concentration. Any sample that did not fall within the standard curve was diluted and reanalyzed. A serum pool of known Hp concentration was analyzed with each assay. Calcium (C294-06, Catachem) [28], cholesterol (C104-02, Catachem) [29,30], Mg (C355-01, Catachem) [31], and Phos (V274-12, Catachem) [32] were also analyzed in serum on the Catachem Well-T AutoAnalyzer (Catachem) in duplicate. A weekly three-point standard curve was generated using the Catacal, Catarol I, and Catatrol II standards (Catachem) for these biomarkers. Inter-assay coefficient of variation was 4.23% 5.52%, 9.51%, 9.23%, and 1.70% for calcium, cholesterol, Hp, magnesium, and phosphorous, respectively; no intra-assay coefficient of variation exceeded 10%.

In both experiments, liver samples (~750 mg) were obtained by blind percutaneous biopsy utilizing a custom-built trocar at 1 and 14 DIM [33,34]. Biopsy samples were immediately rinsed with saline, aliquoted into tubes, frozen in liquid nitrogen, and stored at −80 °C until analysis of liver TG content. Liver TG content was quantified by colorimetric assay as described previously by Folch-extracted product and expressed as a % of DM in the original publications [26,35,36].

### 2.2. Preparation of Data Sets

Liver TG, blood energy metabolite, mineral, and protein biomarker data from the previous experiments were aligned into two data set categories: longitudinal (LT) and single timepoint (ST). For all data sets, each cow was represented as a single observation. The LT data set observations (*n* = 65 cows) had biomarker concentrations from multiple timepoints (1, 3, 5, 7, and 14 DIM) included as explanatory variables for each individual cow. Meanwhile, the ST category data sets had biomarker concentrations from a single DIM as the explanatory variables for each observation. Three ST data sets were created: 3 DIM (ST3, *n* = 65 cows), 7 DIM (ST7, *n* = 65 cows), and 14 DIM (ST14, *n* = 65 cows). The prediction model response variables were consistent across the LT and ST data sets and defined based on a cow’s maximum observed liver TG content (*n* = 11 cows on 1 DIM, *n* = 52 cows on 14 DIM, *n* = 2 cows not assigned due to a missing liver tissue biopsy sample). The only continuous response was maximum liver TG content, natural log transformed (Max TG); the natural log transformation was used due to a modest improvement in model performance (data not presented). In the absence of a well-defined liver TG threshold, three binary responses were explored using percentile thresholds: Low TG (maximum liver TG > 13.3% DM, 33rd percentile), Median TG (maximum liver TG > 17.1% DM, median), and High TG (maximum liver TG > 22.0% DM, 66th percentile). A cow with a maximum liver TG above the respective threshold was coded as the event for that response variable.

The eligible explanatory variables varied across data sets due to differences in the blood biomarkers analyzed within a particular DIM. Parity number, BHB, Ca, cholesterol, FA, glucose, Mg, and Phos were available for all ST data sets. Albumin, ALT, AST, AST:ALT, BUN, and Hp concentrations were available for the ST3 and ST14 data sets. The LT data set included all the previous measurements on their respective DIM (3, 7, and 14 DIM) as separate explanatory variables. In addition, measurements of BHB, Ca, cholesterol, FA, glucose, Mg, and Phos were available on 1 and 5 DIM for the LT data sets; albumin, ALT, AST, AST:ALT, BUN, and Hp were available on 1 DIM for the LT data set. Trapezoidal area under the curve (tAUC) was determined from 3 to 7 DIM for glucose, FA, and BHB concentrations and included as explanatory variables for LT models. Descriptive statistics of the explanatory variables are provided in Table 1. In all data sets, explanatory variables were centered and scaled within DIM by their arithmetic mean and standard deviation, respectively, as derived from the composite data from across the 2 original experiments.

The prediction modeling procedures used in this research are not tolerant of missing data; therefore, a three-step filtering procedure was used to exclude variables and observations (cows) in a manner that maximized the number of useable observations for model training and fitting. Data handling from this point was done in R (version 4.1.0) [37], and filtering was performed on each data set (LT, ST3, ST7, and ST14) independently. First, the potential explanatory variables with >20% missing values within experiment 1 or experiment 2 were excluded from the data sets. Excluded variables included albumin, ALT, AST, AST:ALT, BUN, FA, and Hp, quantified on 1 DIM, as well as the tAUC for FA. 

Second, the variables remaining after step 1 were filtered based on variable importance projection (VIP) scores within a data set (LT, ST3, ST7, or ST14). To get the VIP scores, observations with missing data for the remaining explanatory variables were temporarily omitted to create a complete data set with no missing values (*n* = 44 cows). Then, partial least squares (PLS) modeling methods (mixOmics, version 6.16.0) [38,39] were used to fit an initial prediction model for each response variable (*n* = 4 models per data set). Max TG was predicted by PLS regression; PLS-discriminate analysis (PLSDA) was used for Low TG, Median TG, and High TG models. These initial models were fitted to 3 principal components and VIP scores were extracted for all 3 principal components. Based on the VIP scores generated in the initial modeling, explanatory variables were assigned their maximum observed VIP score across all models and model principal components within a respective data set (LT, ST3, ST7, or ST14). Then, potential explanatory variables with a maximum observed VIP < 1.0 were excluded from the respective data set. 

In the final filtering step, observations that were temporarily omitted in step 2 were added back into the data set as long as they had complete data for the explanatory variables remaining after VIP filtering in step 2. After filtering explanatory variables (step 1, missingness; step 2, VIP scores) and observations with incomplete data (step 3), the LT and ST data sets had *n* = 52 and *n* = 47 observations, respectively, for the subsequent model training.

### 2.3. Model Training, Evaluation, and Validation

Prediction of Max TG, Low TG, Median TG, and High TG was performed by sparse PLS methods (mixOmics). This method of prediction was chosen because it is more robust to multicollinearity than multiple regression methods and allows for the maximization of model performance while using the least number of explanatory variables within a principal component. The latter reason minimizes the number of biomarkers an end user would need to assay for a selected model. Max TG was predicted by sparse PLS regression, while the binary responses were predicted by sparse PLSDA. Models are presented as response variable-data set combinations. For example, a PLS model predicting Max TG using LT data would be designated Max TG-LT and a PLSDA model predicting High TG with ST14 data would be designated High TG-ST14.

The tune.spls() and tune.splsda functions of the mixOmics package were used to exhaustively train models for Max TG and the binary responses, respectively, on all filtered data sets. To exhaustively search different model fittings across varying data, random split cross-validation (RCV) was performed. The RCV used 4 folds and 1000 iterations to explore models fitting 1 to the maximum allowed principal component (explanatory variable count—1) and using 1 to all available loadings within a principal component. The optimum sparse model fitting within a data set was selected based on minimization of mean squared error (MSE) and balanced error rate (BER) for sparse PLS and sparse PLSDA models, respectively. Additional model evaluation statistics were extracted for the optimal models, including area under the receiver operating characteristic curve (rAUC, sparse PLSDA), mean absolute error (MAE, sparse PLS), and coefficient of determination (R^2^, sparse PLS). Then, block cross-validation (BCV) of the optimal models was performed to suggest whether optimal model prediction was dependent on the underlying data structure and resulted in overoptimistic model evaluation [40,41]. Each experiment-treatment combination was considered as a block (*n* = 4 blocks). Briefly, the optimal model was iteratively fit to 3 blocks and used to predict responses of the fourth block, such that all blocks were predicted by the other 3 blocks once. Model evaluation statistics for BCV of sparse PLS models were computed for the aggregate predictions using the Model Evaluation System (version 3.2.4) [42], including root MSE (RMSE), MAE, R^2^, concordance correlation coefficient (CCC) [43], and mean bias. For the BCV sparse PLSDA models, the BER, accuracy, sensitivity, specificity, positive predictive value (PPV), and negative predictive values were calculated [44] using custom R scripts. Within each binary, the classification with greater maximum liver TG was considered the event/case for calculating these statistics. Finally, the final optimal models were fitted to the entire filtered data set so that variable loadings could be extracted and reported (Supplementary Tables S2 and S3).

To increase accessibility of the High TG models, we further explored models post hoc based on what information we conceived to be readily available in existing research data sets or diagnostic laboratories. Two potential barriers to application of the proposed models are the collection of 1 DIM samples and uncommonly analyzed analytes. In our data set, blood fraction analytes we perceived as uncommonly analyzed in the transition cow literature are Ca, cholesterol, Hp, Mg, and Phos. For example, Hp is not common to diagnostic laboratories or diagnostic equipment common to clinicians (examples: i-STAT series, Abbott, Lake Forest, IL, USA; Carysta HVC, Diasys Diagnostic Systems, Wixom, MI, USA; cobas analyzer series, Roche Diagnostics, Indianapolis, IN, USA) and minerals are not always analyzed pre- and postpartum. Thus, we explored four levels of explanatory variable restriction: no 1 DIM data (subscript no1DIM), no Hp measurements (subscript noHp), no Hp or cholesterol measurements (subscript limit1), and no Hp, cholesterol, or minerals (subscript limit2). If the initial sparse PLSDA model for High TG did not include an uncommon analyte (Supplementary Tables S2 and S3), the variable restriction was not explored (i.e., High TG-LT_noHp_, Supplementary Table S2).

## 3. Results and Discussion

Together with improvements in nutrition and management, the increased availability of diagnostic tools and proactive health monitoring have allowed for improvements in postpartum cow health [9]. Hyperketonemia is a hallmark example with several cowside diagnostic tools, including blood BHB quantification with a handheld meter [10,45,46], as well as the development of predictive analytic tools to screen or diagnose cows with hyperketonemia using routinely collected data [47,48,49,50]. Monitoring liver TG content and bFLS has remained elusive to dairy farmers, dairy consultants, and clinicians because doing so requires an invasive liver tissue biopsy, as well as laboratory analysis for TG content to definitively diagnose cases [2]. These challenges have prevented in-depth investigation into the incidence, risk factors, consequences, and financial impact of bFLS. Our novel investigation explored utilizing postpartum concentrations of a variety of blood energy metabolite, protein, and mineral biomarkers in a multivariate prediction of liver TG concentration and binary classifications of TG content in multiparous Holstein dairy cows. We attempted to improve the practicality of these liver TG prediction models by developing several variations based on LT or ST blood sampling, so that these models can be implemented in a broad range of applications. Defining an appropriate bFLS threshold is challenging because there is a lack of data interrogating liver TG content with cow performance or health outcomes. This is further complicated by the variation in the DIM of tissue sample collection and how steatosis is assessed (i.e., histology, lipid vs. TG content, wet vs. DM vs. DNA basis) [5,8,51,52]. With greater liver TG content generally associated with negative consequences, we chose to use the maximum observed liver TG content between the 1 and 14 DIM liver tissue biopsy samples as the basis of our response variables. Most cows in our data set (83%) had the greatest liver TG content at 14 DIM. This is in agreement with previous literature that indicated liver TG content typically peaks between 1 and 3 weeks postpartum [1,53].

Prediction of maximum liver TG as a continuous response is theoretically the most flexible for long term application of a bFLS diagnosis and screening model, and could be of value if a subclinical threshold is established. Across the ST and LT data sets, Max TG prediction models’ mean MSE ranged from 0.66 to 0.89 ln(liver TG, % DM)^2^, mean MAE ranged from 1.12 to 1.4 ln(liver TG, % DM), and R^2^ ranged from 0.11 to 0.33 during RCV (Table 2). Generally, the RMSE, MAE, and R^2^ performance was marginally better for Max TG models during BCV (Table 2), suggesting the model performance was not dependent on the treatment structure [40,41]. Model CCC ranged from 0.17 to 0.43 and mean bias ranged from -0.05 to approximately 0 during BCV across the ST and LT models for Max TG. The models herein are unique in the biomarkers used and the multivariate approach employed to predict liver TG content in dairy cows, so there is no direct comparison of model performance available to benchmark the efficacy of these PLS models. 

Binary classifications can be useful to assess herd metabolic health by suggesting the proportion of sampled cows with relatively low or high liver TG. Indeed, the proportion of early lactation cows with a particular disease, such as hyperketonemia or hypocalcemia, has been used to monitor herd performance and responses to changes in management [9,47]. In the absence of a bFLS diagnostic threshold, the bottom tercile, median, and top tercile of the observed maximum TG were used as thresholds to classify cows as having a greater liver TG than a normal or relatively healthy early lactation cow. A recent investigation associated liver TG content at 8 DIM to cow performance, suggesting that cows with liver TG > 7.0% on a wet weight basis had a detrimental impact on productivity, fertility, and morbidity [8]. That work reported median liver TG content for the data set on a wet and DM basis; the ratio of these medians was 0.348, wet basis:DM basis [8]. Assuming the wet basis:DM basis ratio is constant across samples of varying TG content and DIM, the Low, Median, and High TG thresholds used to classify cows in the current study would approximate 4.6, 6.0, and 7.7% TG on a wet weight basis, respectively. Based on the above noted performance outcomes negatively associated with liver TG > 7.0% wet weight basis [8], the High TG response might best represent a meaningful subclinical bFLS threshold associated with negative consequences. Further epidemiological research is needed to validate these classifications and the impact of bFLS on dairy cow performance [54,55].

There was substantial variation in the performance of sparse PLSDA models that predicted the different binary responses. During RCV, Low TG models had BER ranging from 32.2% to 42.3% and rAUC ranging from 62.9 to 69.5% (Table 3). Classification statistics were also relatively low during BCV of ST models for Low TG [44]; however, Low TG-LT performed better during BCV: 15.7% BER, 80.9% accuracy, 92.4% sensitivity, and 76.5% specificity (Table 3). 

The Median TG-ST3 and Median TG-ST13 models had BER_RCV_ > 33.0% and rAUC_RCV_ < 70.0% for median TG (Table 3). Meanwhile, Median TG-ST7 and Median TG-LT models had rAUC_RCV_ of 80.9 ± 1.2% and 80.2 ± 4.5%, respectively; BER_RCV_ was 19.6 ± 2.4% and 24.0 ± 4.4% for Median TG-ST7 and Median TG-LT, respectively (Table 3). The Median TG-ST7 model performed similarly during BCV with 18.4% BER, 66.7% sensitivity, and 94.2% PPV; Median TG-LT had reduced performance during BCV with 25.3% BER, 57.2% sensitivity, and 85.8% PPV (Table 3). 

**Figure 1:**
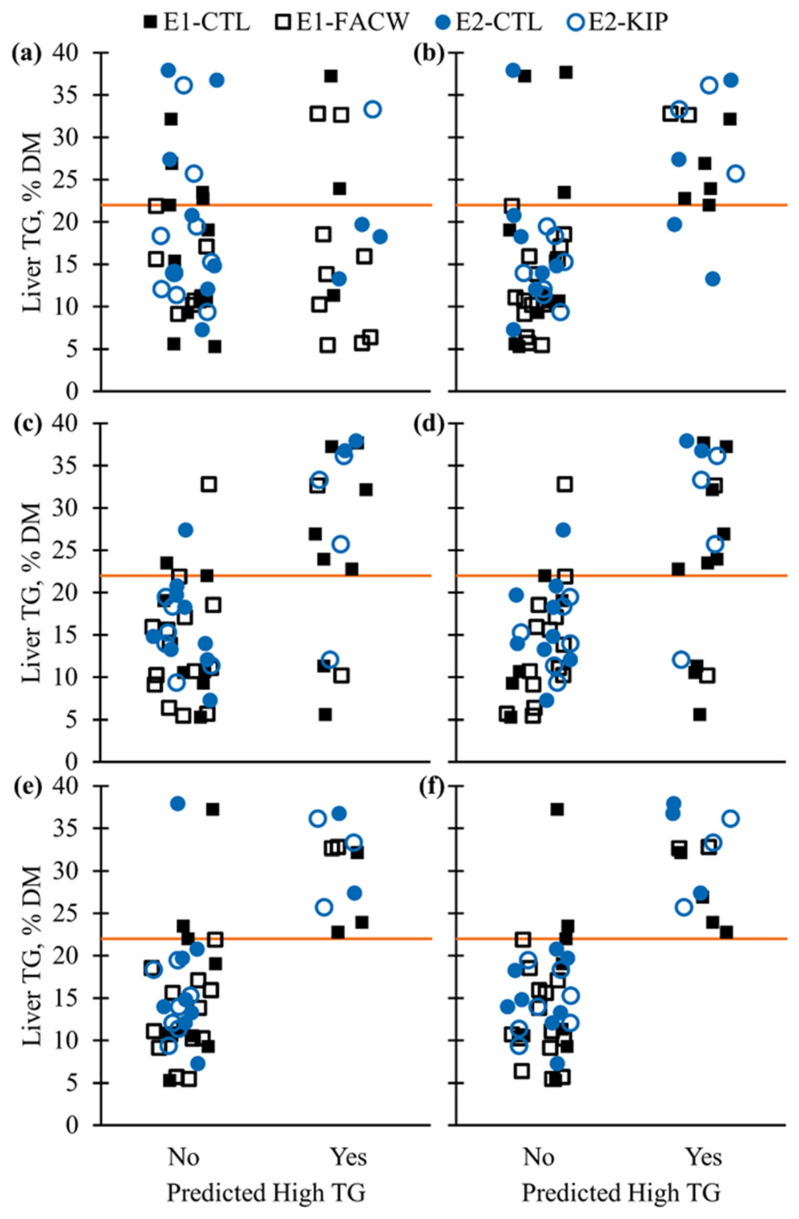
Maximum observed liver tissue triglyceride (TG) content versus High TG status prediction during block cross-validation of sparse partial least squares—discriminate analysis models using blood energy metabolite, protein, and mineral biomarkers. Models used single timepoint (ST, *n* = 52 cows) or longitudinal blood sampling (LT, *n* = 47 cows): (**a**) ST 3 days in milk (DIM), (**b**) ST 7 DIM, (**c**) ST 14 DIM, (**d**) ST 14 DIM without haptoglobin data, (**e**) LT, and (**f**) LT without 1 DIM data. Symbols refer to a cow’s original dietary treatment blocks for experiment 1 (control, E1-CTL; fermented ammoniated condensed whey supplementation, E1-FACW) and experiment 2 (control, E2-CTL; ketosis induction protocol, E2-KIP). The orange line represents the observed liver TG % dry matter (DM) threshold for high TG classification (liver TG > 22.0% DM).

High TG prediction models generally had better performance than the other binary responses, except for the High TG-ST3, High TG-ST14_limit1_, and High TG-ST14_limit2_ models. These lower performing High TG models had BER from 26.6% to 33.8% during RCV (Table 3 and Table 4). Ranges of BER and rAUC during RCV were from 13.3% to 15.4% and from 90.2% to 95.0%, respectively, for High TG prediction models using ST7, ST14, ST14_noHp_, LT, and LT_no1DIM_ data sets. During BCV, High TG-LT_no1DIM_ generally performed better during RCV than other models with 7.2% BER_BCV_, 96.0% accuracy, 85.7% sensitivity, 100.0% PPV, and 100.00% specificity. The High TG models using ST7, ST14, and ST14_noHp_ data had relatively similar performance; while High TG-ST7 performed slightly better during RCV and High TG-ST14_noHp_ had marginally better performance during BCV (Table 3 and Table 4). Performance of the High TG prediction models across the original dietary treatments during BCV can be visualized in Figure 1.

Overall, High TG models generally performed better than Low TG and Median TG models, with Low TG having the lowest performance. When compared to recommendations in the literature, several High TG models achieved a very desirable level of performance during cross-validation with balanced accuracy (100—BER) and rAUC values > 85% [44]. Therefore, the High TG models are most likely to be useful for herd-level surveillance of liver TG content and possibly individual cow decision making [44,48,49]. As with all novel prediction tools, the liver TG prediction models developed as part of this research would benefit from further validation on novel data sets. Future research implementing these models on a larger number of cows from varying environmental conditions may provide indirect evidence of their generalizability through epidemiological assessment of cow productivity and health outcomes [47,50].

Maximizing convenience is a major consideration when developing a tool for implementation in agricultural practices [56,57]. The labor and time required to collect blood samples is a major barrier to monitoring metabolic health in dairy cows that researchers and clinicians have attempted to minimize [9,25,47,56,58]. To that end, an objective of this research was to explore ST for predicting liver TG content, which would be more convenient than LT models on-farm. Caution must be exercised when comparing ST and LT models in this work, especially when comparing BCV statistics that had a lower level of replication, because their cross-validation occurred on different subsets of the data. For prediction of Max TG, Low TG, and Median TG, ST3 and ST14 models appeared to lower performance compared to the ST7 and LT models. The respective ST7 and LT models performed similarly within the Low TG and Median TG response. When interrogating sparse PLS and PLSDA model loadings, the ST7 variables tend to be the only or largest magnitude loadings retained in the respective LT model for those responses and appeared to be driven by blood FA concentration (Supplementary Tables S2 and S3). Comparison of High TG prediction models showed similar results, except that the ST14 models (other than ST14_limit1_ and ST14_limit2_) also performed similar to the LT based models. These comparisons suggest that assessment of maximum liver TG content during early lactation is possible by analysis of a single blood sample at a key DIM timepoint. The 7 and 14 DIM timepoints appeared to perform similar to multiple timepoint models, which corresponds to the approximate DIM window that maximum liver TG accumulation typically occurs [1,53].

To further increase the accessibility of the High TG models, we explored alternative variations with a restricted subset of potential explanatory variables on the high TG models. Two potential barriers to application of the proposed models are the collection of 1 DIM samples and uncommonly analyzed analytes. When comparing High TG-LT to the variable-restricted High TG-LT_no1DIM_, High TG-LT_limit1_, and High TG-LT_limit2_ models, they all performed similarly during cross-validation (Table 3 and Table 4). However, they varied widely in the number of analytes incorporated in their sparse PLSDA models, with 5, 23, 4, and 1 analyte included in the High TG-LT, High TG-LT_no1DIM_, High TG-LT_limit1_, and High TG-LT_limit2_ models, respectively (Supplementary Table S2). The High TG-ST14 and High TG-ST14_noHp_ models had similar performance during RCV and performed better than the High TG-ST14_limit1_ and High TG-ST14_limit2_ models (Table 3 and Table 4). These variations of the High TG-ST14 models had 6 to 8 analytes retained in the sparse PLSDA models (Supplementary Table S3). Based on these observations, data from 1 DIM and Hp data are not essential to maintain High TG-LT model performance; however, removing 1 DIM data resulted in many more biomarkers retained in the PLSDA model. This trade-off between sampling at 1 DIM and LT panel financial cost may result in the High TG-LT_no1DIM_ model being less viable in most scenarios. Meanwhile, the High TG-ST14 and High TG-ST14_noHp_ models performed similarly during cross-validation with great performance and maintained similar numbers of unique biomarkers, suggesting Hp is not an essential component to the ST models predicting High TG.

Biomarkers used within the present study were primarily selected based on biological or epidemiological connections with bFLS; however, candidate biomarkers were censored when they required enzyme-linked immunosorbent assay, radioimmunoassay, or other assays with relatively limited accessibility to ensure that the final models could be implemented by end-users. Although the current study was not designed to make inferences on the relationship of the biomarkers analyzed and bFLS, the biomarkers most often retained in final models were not surprising given what is known about bFLS onset in dairy cattle. For example, blood concentrations of FA, BHB, and glucose were often retained in 19, 11, and 12 of the 22 final prediction models, respectively (Supplementary Tables S2 and S3). Given the interrelationships between the TCA cycle, ketogenesis, gluconeogenesis, and liver TG accumulation in dairy cows, it is logical these biomarkers would be frequently retained predictors of liver TG and bFLS [1,4,23,59]. Either independently or in ratio, AST and ALT were included in all ST3 and ST14 models (Supplementary Table S3). In humans, ALT, AST, and other blood proteins have been used for diagnosis of human steatosis and potential liver injury [60]. Of the minerals quantified, Mg was the most frequently included in the final models (7 of 22 models, Supplementary Tables S2 and S3). Recent research has suggested that Mg has a role in lipogenesis and esterification in cultured bovine adipocytes [61]. With the inclusion of Mg in our prediction models, the potential mechanistic role of Mg in bFLS onset may justify further examination.

Overall, our findings suggest that blood-based panels of postpartum biomarkers can identify early lactation liver TG content, at least in multiparous Holstein cows. This finding generates an exciting opportunity for researchers and industry professionals (i.e., farmers, consultants, and clinicians) to monitor and troubleshoot bFLS in research and field settings where liver tissue biopsy is impractical. The postpartum nature of the blood biomarker analysis in this research implies that these panels would serve as a diagnostic tool and thus act as a retrospective assessment of cow management. In contrast, a panel based on prepartum indicators would allow for proactive intervention and prevention of bFLS. Previous research interrogating prepartum biomarkers to predict liver TG content and illness have suggested the potential for development of such a tool but unfortunately, the data was not available in the present study to interrogate that potential. Furthermore, the opportunity to use one or more ST models on farm presents the potential to evaluate liver TG content as a part of troubleshooting or health management. The potential impact of High TG status on production and the role of nutritional or therapeutic interventions still needs to be explored in larger, epidemiological studies [54,56,62,63]; however, presence of tools such as the blood biomarker panels presented herein will make these studies more practical.

## 4. Conclusions

This research represented a novel investigation into a multivariate approach to predict liver TG content in early lactation dairy cows. Prediction of maximum liver TG content as a continuous variable did not perform as well as prediction models that have been validated for other metabolic health prediction. Models predicting Low TG (TG > 13.3% liver DM) and Median TG (TG > 17.1% liver DM) thresholds had relatively low performance. In contrast, prediction of High TG (TG > 22.0% liver DM) had desirable accuracy when using biomarker data at 7 DIM, 14 DIM, and LT sampling. Based on model performance and preliminary comparison of our models to association of liver TG content to adverse consequences, the High TG threshold models may be a useful diagnostic or monitoring tool for bFLS in research and field settings.

## Figures and Tables

**Table 1:** Descriptive statistics of liver triglyceride (TG) and potential explanatory variables in the composite data set ^1^ before filtering procedures ^2^.

Variable ^3^	DIM ^4^	*n*	Mean	SD	Min	Q1	Median	Q3	Max
Parity		64	3.08	1.25	2.00	2.00	3.00	4.00	7.00
Liver TG, % DM	1	63	9.15	5.74	2.64	5.36	7.97	10.75	37.95
	14	62	17.78	10.61	2.21	9.39	15.34	24.40	45.85
	Max	62	18.95	10.32	5.28	10.97	17.10	25.83	45.85
ln(Liver TG, % DM)	Max	62	2.79	0.57	1.66	2.40	2.80	3.25	3.83
Blood Biomarkers									
Glucose, mg/dL	1	64	67.19	18.78	43.36	57.77	61.27	70.02	143.54
	3	64	55.86	7.05	40.85	51.18	55.65	60.02	81.06
	4	62	54.23	7.40	33.06	50.12	54.90	59.25	70.12
	5	63	53.53	7.17	29.43	49.41	53.87	59.04	66.26
	14	64	53.64	6.17	38.93	49.62	54.32	58.62	64.79
	tAUC	62	341.67	38.18	252.96	311.38	346.08	368.43	430.08
Fatty acids, mEq/L	1	38	0.49	0.24	0.20	0.34	0.43	0.58	1.32
	3	63	0.50	0.23	0.09	0.36	0.45	0.65	1.05
	5	63	0.47	0.24	0.12	0.30	0.45	0.57	1.40
	7	60	0.42	0.22	0.09	0.28	0.35	0.54	1.14
	14	62	0.40	0.21	0.13	0.24	0.37	0.52	1.24
	tAUC	33	2.97	1.13	1.51	2.11	2.62	3.70	6.19
BHB, mM	1	63	0.60	0.18	0.30	0.49	0.58	0.68	1.16
	3	64	0.83	0.37	0.35	0.60	0.76	0.91	2.17
	5	63	0.91	0.66	0.41	0.62	0.76	0.94	4.03
	7	64	0.95	0.71	0.36	0.63	0.74	0.97	5.42
	14	64	0.90	0.44	0.37	0.66	0.78	0.94	2.47
	tAUC	63	5.03	2.53	2.57	3.73	4.34	5.45	17.54
Albumin, g/dL	1	36	3.85	0.23	3.44	3.71	3.81	3.99	4.48
	3	64	3.74	0.23	3.08	3.61	3.76	3.88	4.24
	14	57	3.89	0.31	3.27	3.61	3.95	4.14	4.48
ALT, U/L	1	37	18.92	6.46	6.87	14.50	18.62	22.35	37.82
	3	64	14.29	4.56	5.70	10.51	14.16	18.02	25.26
	14	62	15.98	5.48	7.51	11.70	15.41	19.88	34.11
AST, U/L	1	40	73.74	22.09	40.28	52.76	74.29	89.73	119.04
	3	64	82.97	29.77	38.31	62.49	78.77	93.94	209.51
	14	63	93.73	39.46	44.08	73.05	83.93	103.67	308.58
AST:ALT	1	37	4.12	1.20	2.39	3.16	4.12	4.96	7.08
	3	64	6.36	3.01	3.50	4.15	5.21	7.73	17.42
	14	62	6.47	3.19	2.52	3.98	5.67	8.09	15.83
BUN, mg/dL	1	39	11.38	2.90	5.98	8.88	11.67	13.35	18.61
	3	64	11.22	2.93	6.50	8.96	10.86	12.98	20.50
	14	63	13.33	2.87	7.19	11.12	13.36	15.00	20.33
Hp, mg/dL	1	38	0.76	0.51	0.11	0.36	0.65	0.96	2.69
	3	63	2.04	1.39	0.22	0.87	1.74	2.85	6.22
	14	62	0.51	0.49	0.06	0.23	0.32	0.56	2.83
Ca, mg/dL	1	50	6.76	1.00	4.50	6.31	6.78	7.38	9.20
	3	52	7.99	1.02	4.35	7.56	8.15	8.70	9.70
	5	52	8.41	1.07	5.15	7.61	8.35	9.35	10.85
	7	53	8.21	1.16	4.85	7.45	8.45	8.88	11.20
	14	52	8.65	0.82	6.45	8.28	8.73	9.25	10.05
Mg, mg/dL	1	51	2.15	0.38	1.39	1.83	2.15	2.37	3.22
	3	53	2.18	0.30	1.10	2.04	2.19	2.35	2.94
	5	53	1.89	0.33	1.01	1.67	1.88	2.12	2.52
	7	53	1.89	0.30	1.17	1.69	1.88	2.09	2.50
	14	52	2.20	0.38	1.26	2.02	2.24	2.53	2.87
Phos, mg/dL	1	51	3.79	1.05	1.63	3.11	3.59	4.46	6.06
	3	53	4.48	1.07	1.95	3.85	4.40	4.97	8.15
	5	53	4.49	0.88	2.16	3.93	4.46	5.16	6.01
	7	53	4.07	0.65	2.76	3.55	4.04	4.60	5.46
	14	52	4.28	0.80	2.66	3.70	4.18	4.80	5.89
Cholesterol, mg/dL	1	51	59.30	11.87	38.46	49.75	58.92	65.27	92.08
	3	53	63.81	12.28	34.68	54.55	64.30	71.71	96.81
	5	53	68.86	12.81	41.28	59.79	69.00	74.79	97.54
	7	53	77.05	13.51	45.88	67.37	75.86	85.80	105.48
	14	52	112.89	20.72	79.47	97.90	108.92	128.87	169.06

^1^ The composite data set included multiparous Holstein dairy cows (*n* = 65) from two separate experiments with two experimental treatments per experiment, ^2^ Table heading definitions: DIM = day in milk timepoint, *n* = sample size, SD = standard deviation, Q1 = quartile 1 threshold, Q3 = quartile 3 threshold, ^3^ Abbreviations used in rows to describe variables: DM = dry matter, BHB = β-hydroxybutyrate, ALT = alanine transanimase, AST = aspartate transanimase, BUN = blood urea nitrogen, Hp = haptoglobin, Ca = calcium, Mg = magnesium, Phos = phosphorous, ^4^ Abbreviations used DIM column: Max = max observed value from 1 to 14 DIM, tAUC = trapezoidal area under the curve from 3 to 7 DIM.

**Table 2:** Model evaluation statistics from the cross-validation (CV) of sparse partial least squares models that predict max liver triglyceride (TG) content ^1^.

Variables ^3^	Random Split CV ^4^						Block CV ^8^				
	MSE ^5^		MAE ^6^		R^2^						
	Mean	SE ^7^	Mean	SE	Mean	SE	RMSE	MAE	R^2^	CCC ^9^	Mean Bias
ST3	0.81	0.07	1.40	0.06	0.20	0.05	0.47	0.39	0.25	0.28	−0.01
ST7	0.70	0.05	1.20	0.04	0.29	0.04	0.45	0.35	0.34	0.36	<0.01
ST14	0.89	0.04	1.40	0.04	0.11	0.03	0.52	0.43	0.12	0.17	−0.05
LT	0.66	0.05	1.20	0.05	0.33	0.05	0.44	0.35	0.38	0.43	−0.01

^1^ Liver TG (% liver tissue dry matter) was assessed at 1 and 14 days in milk (DIM) for every cow. The natural log transformation of the maximum liver TG observed across DIM was used as the response variable for all models. ^2^ Coefficient of determination, ^3^ Explanatory variables included blood concentrations of energy metabolite, protein, and mineral biomarkers. Models varied in biomarker availability based on single timepoint (ST) or longitudinal (LT; multiple timepoint) blood sampling. Day in milk of ST models (*n* = 52 cows) were 3, 7, and 14 DIM for ST3, ST7, and ST14, respectively. The LT models (*n* = 47 cows) could include data from 1, 3, 5, 7, and 14 DIM. ^4^ Random split CV of data using 4 folds and 1000 replications, ^5^ Mean squared error, ^6^ Mean absolute error, ^7^ Standard error, ^8^ Original dietary treatments (*n* = 4, 2 experiments) alternated as folds during CV, ^9^ Concordance correlation coefficient.

**Table 3:** Model evaluation statistics from the cross-validation (CV) of sparse partial least squares—discriminate analysis models that predict binary classification of liver triglyceride (TG) content.

Model		Random Split CV ^3^				Block CV ^7^					
		BER ^4^		rAUC ^5^							
Response ^1^	Explanatory ^2^	Mean	SE ^6^	Mean	SE	BER	Accuracy	Sensitivity	Specificity	PPV ^8^	NPV ^9^
Low TG	ST3	35.7	5.2	69.5	5.1	43.1	53.9	45.5	68.5	28.6	74.2
	ST7	32.2	2.1	75.7	1.2	28.5	68.0	58.9	84.3	87.0	53.4
	ST14	42.3	3.7	63.0	4.1	40.8	59.7	60.7	57.9	71.5	45.9
	LT	33.9	6.4	69.2	6.0	15.7	80.9	92.4	76.5	60.0	96.3
Median TG	ST3	33.8	4.3	69.9	3.9	33.0	67.4	65.3	69.0	37.5	82.2
	ST7	19.6	2.4	80.9	1.2	18.4	83.1	66.7	96.6	94.2	77.8
	ST14	37.9	5.0	61.8	4.8	48.9	52.0	41.7	60.8	47.7	54.9
	LT	24.0	4.4	80.2	4.5	25.3	76.6	57.2	92.4	85.8	72.8
High TG	ST3	35.4	3.5	68.2	3.8	46.7	61.6	35.8	71.1	31.3	75.0
	ST7	13.9	2.5	95.0	0.8	17.3	86.8	73.4	92.2	78.6	89.8
	ST14	15.3	3.0	90.5	1.9	15.5	86.6	80.0	89.2	75.0	91.7
	LT	15.4	3.9	90.8	3.7	11.6	93.7	77.0	100.0	100.0	91.9

^1^ Binary classifications are assessed based on the maximum observed liver TG at 1 or 14 days in milk (DIM), with events (or cases) defined as being above a liver TG threshold (% liver tissue dry matter; DM). Response variable thresholds were: Low TG > 13.3% DM, Median TG > 17.1% DM, and High TG > 22.0% DM. ^2^ Explanatory variables included blood concentrations of energy metabolite, protein, and mineral biomarkers. Models varied in biomarker availability based on single timepoint (ST) or longitudinal (LT; multiple timepoint) blood sampling. Day in milk of ST models (*n* = 52 cows) were 3, 7, and 14 DIM for ST3, ST7, and ST14, respectively. The LT models (*n* = 47 cows) could include data from 1, 3, 5, 7, and 14 DIM. ^3^ Random split CV of data using 4 folds and 1000 replications, ^4^ Balanced error rate, ^5^ Area under the receiver operating characteristic curve, ^6^ Standard error, ^7^ Original dietary treatments (*n* = 4, 2 experiments) alternated as folds during CV, ^8^ Positive predictive value, ^9^ Negative predictive value.

**Table 4:** Model evaluation statistics from the cross-validation (CV) of sparse partial least squares—discriminate analysis models that predict high liver triglyceride (TG) content using explanatory variables based on perceived accessibility ^1^.

Model		Random Split CV ^3^				Block CV ^7^					
		BER ^4^		ROC AUC ^5^							
Sampling	Explanatory ^2^	Mean	SE ^6^	Mean	SE	BER	Accuracy	Sensitivity	Specificity	PPV ^8^	NPV ^9^
ST14	noHp	15.3	3.1	90.2	1.8	13.4	86.5	86.7	86.5	72.2	94.1
	limit1	26.6	4.5	80.5	3.5	27.1	76.9	73.3	78.4	57.9	87.9
	limit2	24.3	2.8	84.6	2.1	20.7	86.2	61.1	97.5	91.7	84.8
LT	no1DIM	13.3	3.2	94.3	2.7	7.2	96.0	85.7	100.0	100.0	94.7
	limit1	15.3	5.0	91.5	4.1	15.4	91.5	69.2	100.0	100.0	89.5
	limit2	17.6	3.6	86.5	2.7	18.3	86.8	68.8	94.6	84.6	87.5

^1^ High TG classification was assessed based on the maximum observed liver TG at 1 or 14 days in milk (DIM), with events (or cases) defined as maximum liver TG > 22.0% liver tissue dry matter. ^2^ Explanatory variables included blood concentrations of energy metabolite, protein, and mineral biomarkers. Models varied in biomarker availability based on single timepoint (ST) or longitudinal (LT; multiple timepoint) blood sampling. Day in milk of ST models (*n* = 52 cows) were 3, 7, and 14 DIM for ST3, ST7, and ST14, respectively. The LT models (*n* = 47 cows) could include data from (except no1DIM) 1, 3, 5, 7, and 14 DIM. The noHp was not allowed to include haptoglobin (Hp) measurements, limit1 models were not allowed to include Hp or cholesterol measurements, and the limit2 models were not allowed to include Hp, cholesterol, or mineral measurements. ^3^ Random split CV of data using 4 folds and 1000 replications, ^4^ Balanced error rate, ^5^ Area under the receiver operating characteristic curve, ^6^ Standard error, ^7^ Original dietary treatments (*n* = 4, 2 experiments) alternated as folds during CV, ^8^ Positive predictive value, ^9^ Negative predictive value.

## Data Availability

Data are available upon reasonable request from the corresponding author.

## Supplementary Materials

The following supporting information can be downloaded at: https://www.mdpi.com/article/10.3390/ani12192556/s1, Table S1: Descriptive statistics of cow production and biometric data by original experimental treatment and liver triglyceride category; Table S2: Explanatory variable principal component (PC) loading values for sparse partial least squares (-discriminate analysis) models using longitudinal (LT) data to predict liver triglyceride (TG) accumulation; Table S3: Explanatory variable principal component (PC) loading values for sparse partial least squares (-discriminate analysis) models using single timepoint (ST) data to predict liver triglyceride (TG) accumulation.
